# Effects of budesonide combined with salbutamol on pulmonary function and peripheral blood eosinophiles and IgE in patients with acute attack of bronchial asthma

**DOI:** 10.12669/pjms.38.6.5718

**Published:** 2022

**Authors:** Jing Liu, Yu Wang, Yanfen Tang, Gang Liu, Qi Chen

**Affiliations:** 1 Jing Liu, Department of Respiratory Medicine, Beijing Ditan Hospital Capital Medical University, Beijing 100015, P.R. China; 2 Yu Wang, Department of Respiratory Medicine, Beijing Ditan Hospital Capital Medical University, Beijing 100015, P.R. China; 3 Yanfen Tang, Department of Respiratory Medicine, Beijing Ditan Hospital Capital Medical University, Beijing 100015, P.R. China; 4 Gang Liu, Department of Respiratory Medicine, Beijing Ditan Hospital Capital Medical University, Beijing 100015, P.R. China; 5 Qi Chen, Department of Respiratory Medicine, Beijing Ditan Hospital Capital Medical University, Beijing 100015, P.R. China

**Keywords:** Acute attack of bronchial asthma, Salbutamol, Budesonide, Pulmonary function

## Abstract

**Objectives:**

To explore the effect and value of salbutamol and budesonide in the treatment of patients with acute attack of bronchial asthma.

**Methods:**

Medical records of patients with acute attack of bronchial asthma treated in our hospital from February 2020 to March 2021 were retrospectively analyzed. Of them, 30 patients received salbutamol atomization inhalation treatment in addition to routine treatment such as cough relief, oxygen inhalation and asthma relief (Group-I), and 40 patients received routine treatment and budesonide and salbutamol atomization inhalation (Group-II). The time of symptom improvement, the improvement in pulmonary function indexes and in peripheral blood eosinophile (EOS) and IgE levels were retrospectively analyzed and compared between the two schemes.

**Results:**

After the treatment, the average time of respiratory restriction, cough, shortness of breath and wheezing improvement in patients, treated with Group-II were lower than those in Group-I (P<0.05). Forced expiratory volume in the first second (FEV1), forced vital capacity (FVC) and peak expiratory flow (PEF) in Group-II were significantly higher than those in Group-I (P<0.05). Post treatment levels of EOS and IgE in peripheral blood of Group-II were significantly lower than those of Group-I (P<0.05).

**Conclusion:**

On the basis of routine treatment, budesonide combined with salbutamol atomization inhalation for the treatment of patients with acute attack of asthma can be more efficient in improving symptoms and pulmonary, function and promote the effective reduction of EOS and IgE levels in peripheral blood.

## INTRODUCTION

Bronchial asthma is a multiple chronic airway inflammatory disease that is characterized by its main pathological feature, airway hyperresponsiveness.^1^ The occurrence of the disease is mainly related to the airway inflammation, allergy and other neuromodulation abnormalities. The main clinical symptoms of patients are shortness of breath, wheezing, chest tightness, repeated cough, etc.^2^,^3^ Bronchial smooth muscle spasm stimulates the increasing secretion of airway mucosa, resulting in swelling of the mucosa and acute asthma attack.^4^ If not timely controlled, the continuous aggravation of the disease can cause respiratory failure and pulmonary heart disease, which seriously affects quality of life and safety of patients. The main goal of the treatment for patients with acute asthma attack is to quickly relieve the respiratory spasm, timely and effectively reverse the hypoxic state of the patient, promote rapid improvement of relevant clinical symptoms, and minimize the damage to lung function.^5^,^6^ At present, patients with acute asthma are mainly treated by using pharmacological agents. However, there is a significant variability in the efficacy of different drugs or schemes. Salbutamol is a commonly prescribed bronchodilator. It is administered by atomization inhalation that can promote rapid entry of drugs into the lesion tissue, improve the drug concentration, exert the inhibitory effect of histamine and other spasmogenic substances, and quickly alleviate the airway spasm and improve the clinical symptoms and signs of patients.^7^,^8^ However, the results of previous studies and clinical practice show that when salbutamol is used alone, its inhibitory effect on airway inflammation is relatively poor. Budesonide is an inhaled glucocorticoid widely used in clinical practice. It can effectively inhibit the secretion of relevant inflammatory mediators during acute attack and has a good inflammatory control effect.^9^ This retrospective study discusses the clinical effect of a combined salbutamol and budesonide atomization inhalation regimen on patients with acute asthma attack and its effectiveness in improving pulmonary function and peripheral blood EOS and IgE levels.

## METHODS

Medical records of 70 patients (48 males and 22 females) with acute attack of bronchial asthma, treated in our hospital from February 2020 to March 2021, were collected. The sample size was estimated by PASS 11, the inspection level was set as a = 0.025 (zero one side), and the inspection efficiency was 1-β= 0.80. Patients were retrospectively divided into two groups based on the received inhalation therapy. Patients (n=30) that were treated with salbutamol atomization inhalation in addition to routine treatment (cough relief, oxygen inhalation and asthma relief) comprised the Group-I. Patients (n=40) that received routine treatment in combination with budesonide and salbutamol atomization inhalation comprised Group-II.

### Inclusion criteria:

• It meets the diagnostic criteria of acute attack of bronchial asthma in the revision of GINA guidelines and the prevention and treatment of bronchial asthma;^10^
• It meets the diagnostic criteria and admission criteria of acute attack of bronchial;
• in acute attack stage;
• Conscious, with normal vision and hearing;
• No systemic steroids were administered orally or intravenously;
• Full medical records related to clinical diagnosis and treatment.

### Exclusion criteria:

• Hematopoiesis, immune system and cardiovascular diseases, serious primary diseases;
• Diagnosed with interstitial lung disease, bronchiectasis, pulmonary tuberculosis and other pulmonary diseases;
• A history of mental illness;
• Contraindications to the drugs used in the study or a history of allergy.

All processes of this study were in full compliance with the relevant rules and regulations of the medical ethics committee of our hospital (Approval number: 2019068-01, Date: 2019-Aug-22).

Routine and salbutamol atomization inhalation therapy was administered using the following protocol: after the admission, the patient received routine medical symptomatic treatment that included oxygen and anti-infection treatment with 250ml 5% glucose injection and Darizone 2.0g intravenous drip; 250ml of 5% glucose injection, 0.5g of diprophylline and 150mg of succinyl hydrocortisone were administered intravenously to relieve spasm and asthma. In addition, patient was given salbutamol sulfate solution (GlaxoSmithKline Australia Pty Ltd, Registration Certificate No.: H20110457, specification:2.5ml: 5mg*5 pieces) for inhalation. Salbutamol (2.5mg) was evenly mixed with 5ml normal saline, and then put into the atomizer storage tank for atomization treatment. Atomization was performed for 10~15 minutes/time, three times/day, and continued for 10 days as a course of treatment.

Routine salbutamol and budesonide atomization inhalation therapy was carried out using the following guidelines: on the basis of routine and salbutamol atomization inhalation therapy, the patient was given inhaled budesonide suspension (AstraZeneca Pty Ltd, Registration Certificate No.: H20140475, specification: 2ml: 1mg * 5 pieces) atomization inhalation therapy. Budesonide (1mg) was evenly mixed in 5ml normal saline and administered by atomized inhalation for 10~15 minutes/time, three times daily for 10 days as a course of treatment.

For all patients, according to the patient’s treatment records, relevant basic data and indicators were collected after receiving corresponding treatment for a course of the treatment. The specific indicators were as follows:

### Symptom regression time

Regression time of symptoms such as shortness of breath, cough and respiratory restriction

### Changes of pulmonary function indexes

measurement results of relevant pulmonary function indexes before and after the treatment (after one course of treatment with corresponding drugs). Lung function was evaluated by German Yeger lung function test system. The specific indexes were one second forced expiratory volume (FEV1), forced vital capacity (FVC) and peak expiratory flow (PEF);

Levels of EOS (eosinophil count) and IgE (immunoglobulin E) in peripheral blood before and after treatment. EOS was counted by automatic blood cell analyzer, and IgE level was detected by enzyme-linked immunosorbent assay (ELISA);

• Incidences of related adverse reactions during treatment.

The relevant data collected were statistically analyzed and processed by spss22.0 software. The measurement data are represented by (*X̅*±s), the normal distribution is represented by t-test, and the non-normal distribution data is represented by rank sum test. Count data are represented by percentage “%”, and the comparison line *Χ^2^*. P<0.05 was considered statistically significant.

## RESULTS

Medical records of 70 patients met the inclusion criteria. Of them, 30 patients received routine treatment and salbutamol atomization inhalation and comprised the Group-I, and 40 patients received routine treatment and budesonide and salbutamol atomization inhalation (Group-II). After statistical analysis, there was no significant difference in gender, age and other general characteristics between the two groups (P>0.05) (Table-I). After corresponding treatment, the average time of relevant symptoms subsided of patients in Group-II were significantly lower than those in Group-I (P<0.05) (Table-II). There was no significant difference in the measurement results of relevant pulmonary function indexes between the two schemes before receiving corresponding drugs. However, after one course of treatment with corresponding drugs, FEV1, FVC and PEF of the patients in both groups significantly improved compared with those before treatment and were significantly higher in the patients in the Group-II compared to Group-I (P<0.05) (Table-III). There was no significant difference in the levels of EOS and IgE in peripheral blood before treatment (P>0.05). After the treatment, levels of EOS and IgE in peripheral blood in Group-II were lower than those in Group-I, and the difference was statistically significant(P<0.05) (Table-IV).

**Table I:** Comparison of two groups of master data.

Group	n	Gender[n]	Age (year,*x̅*±s)	Course of disease (year,*x̅*±s)	Severity of illness		
	
		M/F			Mild	Moderate	Severe
Single drug group	30	18/12	41~75(58.36±11.52)	1~7(3.83±1.57)	11	12	7
Joint group	40	23/17	41~74(57.62±11.17)	1~6(3.55±1.88)	10	17	13
Χ^2^/t	-	0.044	0.271	0.667	1.308		
P	-	0.834	0.787	0.507	0.520		

**Table II:** Comparison of regression time of related clinical symptoms and hospitalization time between the two groups (*x̅*±s).

Group	n	Cough subsides	Wheezing subsided	Resolution of respiratory restriction
Single drug group	30	10.80±1.67	8.86±1.19	5.83±1.72
Joint group	40	8.10±1.51	6.52±1.72	3.72±1.6
t	-	7.062	6.704	5193
P	-	<0.001	<0.001	<0.001

**Table III:** Comparison of several pulmonary function indexes between the two groups (*x̅*±s).

Group	n	FEV_1_ (L)		FVC (L)		PEF (L/s)	

		Before treatment	After treatment	Before treatment	After treatment	Before treatment	After treatment
Single drug group	30	1.82±0.49	2.39±0.57	2.63±0.48	2.92±0.48	2.28±0.72	2.86±0.71
Joint group	40	1.85±0.54	2.87±0.48	2.53±0.62	3.50±0.66	2.36±0.83	3.34±0.81
t	-	0.186	3.840	0.734	4.097	0.443	2.600
P	-	0.853	<0.001	0.465	<0.001	0.659	0.011

**Table IV:** Comparison of EOS and IgE levels in peripheral blood between the two groups (*x̅*±s).

Group	n	EOS in peripheral blood (%)		IgE (IU/L)	

		Before treatment	After treatment	Before treatment	After treatment
Single drug group	30	0.20±0.08	0.15±0.05	105.63±28.22	66.37±14.48
Joint group	40	0.19±0.08	0.08±0.06	106.22±32.67	47.10±10.21
t	-	0.294	4.562	0.079	6.221
P	-	0.770	<0.001	0.937	<0.001

## DISCUSSION

In this study, the relevant clinical data of 70 patients with acute attack of bronchial asthma were collected, and the comparative analysis of different treatment schemes was carried out retrospectively. Our results showed that in patients who received salbutamol and budesonide atomization treatment on the basis of routine treatment (Group-II), the regression time of the main clinical symptoms (shortness of breath, cough, respiratory restriction and wheezing) was significantly shorter than that of patient in Group-I (salbutamol alone). Combined salbutamol and budesonide treatment can significantly reduce the levels of EOS and IgE in peripheral blood. There was no significant difference in the incidence of related adverse reactions between the two regimens. Our results indicate that the combined atomization inhalation of salbutamol and budesonide in the clinical treatment of patients with acute asthma attack can significantly accelerate the improvement of patients’ symptoms. Our results are in agreement with the previous studies. In a double-blind, placebo-controlled trial by Richard B et al.^11^, 668 patients were randomly assigned to one of three treatment groups: salbutamol group, budesonide+salbutamol maintenance group and budesonide+formoterol group. They showed that after 52 weeks of follow-up, the annual asthma deterioration rate in patients in the budesonide+formoterol group was not significantly different, but significantly lower than that of salbutamol group. These results show that budesonide combined with salbutamol is better than salbutamol alone in preventing the deterioration of asthma. Kang Q et al.^12^ studied the efficacy of budesonide atomization inhalation, salbutamol and vitamin D supplementation on asthmatic children. Ninety-six pediatric asthma patients were equally divided into two treatment groups. The patients in the control group were only treated with budesonide, and the patients in the observation group were treated with budesonide atomization inhalation combined with salbutamol and vitamin D supplementation. After the treatment, the levels of IL-2 and IFN-γ in the observation group were significantly higher and levels of IL-4 and IL-6 were significantly lower than those in the control group (P<0.05). Pulmonary functions of the observation group were better than that of the control group (P<0.05). These results showed that budesonide atomization inhalation combined with salbutamol and vitamin D supplementation could significantly improve the inflammatory response in asthmatic children. The above results are consistent with the results of this study.

Salbutamol is a short-acting β2 adrenoceptor agonist that selectively interacts with airway smooth muscle β2 receptor. This interaction can inhibit the release of histamine and other related allergic substances, effectively activate adenylate cyclase activity, promote rapid increase in cyclic adenosine monophosphate level in cells, accelerate the loss of calcium ions and plays the role of relieving bronchospasm.^13^ Using budesonide atomization inhalation to treat asthmatic patients can significantly inhibit the allergic response of the patient’s airway, avoid bronchospasm and improve relevant clinical symptoms. When salbutamol is used alone in the clinical treatment of patients with acute asthma attack, the patients’ asthma, shortness of breath and other related symptoms can be significantly improved, but the effect on patients’ airway inflammation and the overall curative effect and prognosis are limited.^14^

Budesonide is an adrenocortical drug widely used in clinic. The drug has significantly higher binding force to adrenocortical hormone receptor, can significantly interfere with the activation and chemotaxis of eosinophils, effectively inhibit the secretion and synthesis of inflammatory factors. Bateman ED et al.^15^

showed that budesonide has a significant local anti-inflammatory effect. Additionally, it can promote the stability of lysosomal membrane and smooth muscle and endothelial cells and reduce the activity of allergic mediators. Budesonide can also significantly inhibit enzymatic processes stimulated by antigen-antibody binding, and shows a good inhibitory effect on the synthesis and release of bronchoconstrictor substances, thus reducing the contraction of smooth muscle.^16^ At present, it is widely used in the clinical treatment of respiratory diseases and shows a good application effect.^17^ If the patients are treated with budesonide via aerosol inhalation, only a small amount of drug enters the blood stream through the oropharynx, which will not affect the patient’s whole body system. Atomization inhalation allows budesonide to act directly on the lesion and quickly alleviate the symptoms. This mode of treatment promotes better improvement of the patient’s lung function, improves the control of airway inflammation, enhances the stability of respiratory tract, inhibits allergic factors and reduces allergic reaction, improving the overall prognosis.

### Limitations of the study

It is a single center retrospective study with a small sample size, and the patients are over 38 years of age. Further larger multi-center studies that will include pediatric patients, as well as prospective studies, are needed.

## CONCLUSION

The combination of salbutamol atomization inhalation and budesonide atomization inhalation in the treatment of patients with acute attack of bronchial asthma is associated with improved pulmonary function and better anti-inflammatory and anti-allergic effect of the treatment. This study can provide reference for the treatment of patients with acute asthma attack.

### Authors’ Contributions:

**JL:** Conceived and designed the study.

**YW, YT, GL & QC:** Collected the data and performed the analysis.

**JL:** Was involved in the writing of the manuscript and is responsible for the integrity of the study.

All authors have read and approved the final manuscript.
